# Virtual Transition of Care Clinics and Associated Readmission Rates: 3-Year Retrospective Cohort Study

**DOI:** 10.2196/73495

**Published:** 2025-09-23

**Authors:** Sarah F Horman, Milla Kviatkovsky, Edward Castillo, Patricia Maysent, Chad VanDenBerg, John Bell, Christopher A Longhurst

**Affiliations:** 1 Department of Medicine University of California, San Diego San Diego, CA United States; 2 Jacobs Center for Health Innovation University of California San Diego Health San Diego, CA United States; 3 Department of Emergency Medicine University of California, San Diego San Diego, CA United States; 4 Department of Biomedical Informatics University of California San Diego San Diego, CA United States

**Keywords:** transitions of care, telehealth, telemedicine, hospital medicine, quality and patient safety, health care utilization

## Abstract

**Background:**

Hospital readmissions pose a significant burden on patients, health care providers, and systems, with an estimated annual cost of $17 billion. Timely follow-up within 7 days postdischarge is known to reduce readmissions but is often limited by access constraints. While transitions of care clinics have demonstrated benefits in reducing unplanned readmissions, physical space requirements can be logistically and financially challenging.

**Objective:**

This study aimed to evaluate the effectiveness of a virtual transitions of care (VToC) clinic in reducing 30-day hospital readmissions and improving postdischarge care coordination.

**Methods:**

University of California, San Diego Health implemented a hospitalist-led VToC clinic designed to support clinical management, medication reconciliation, primary care provider repatriation, and specialty care navigation. The study included 2314 patients seen in the VToC clinic between September 2021 and September 2024. Outcomes were compared to a benchmark group using regression analysis to assess the impact on 30-day readmission rates.

**Results:**

The 30-day readmission rate for VToC patients was 14.9% (344/2314), significantly lower than the 20.1% (4659/23,129) observed in the benchmark group (*P*<.001). Regression analysis indicated that patients not participating in the VToC clinic had a higher likelihood of readmission (odds ratio=1.37; 95% CI=1.21-1.54; *P*<.001). The most substantial reduction in readmissions was observed among patients with moderate readmission risk (LACE+ score of 50-75).

**Conclusions:**

VToC clinics are a feasible and effective strategy for enhancing postdischarge care, reducing hospital readmissions, and improving care coordination. This model supports the quadruple aim by promoting better health outcomes, improved patient experience, cost-efficiency, and care equity.

## Introduction

Unexpected return to the hospital reflects systemic challenges in health care delivery and places a financial strain on the nation’s health system [1]. Postdischarge follow-ups within 7 days is a well-established protective mechanism to reduce the risk of readmission and mitigate health care disparities but are limited by access challenges and physician shortages [2-5]. Transitions of care clinics have shown benefit in readmission reduction but may be cost prohibitive and logistically challenging [4,5]. Virtual postdischarge clinics may achieve similar benefits with fewer costs and present an opportunity to enhance postdischarge access [6].

## Methods

### Overview

At the University of California, San Diego Health, we implemented a hospital medicine physician-led virtual transitions of care (VToC) clinic, focused on postdischarge clinical management, medication reconciliation, primary care provider (PCP) repatriation, and specialty care navigation, with the aim of reducing repeat hospitalization.

Based on internal pilot data showing a favorable readmission impact for patients with moderate risk of readmission, we created a criteria-based order to trigger referral for patients (aged 18 years and older) discharged from the hospital medicine service line. The discharging providers were automatically prompted at discharge to refer patients to the clinic if patients had a LACE+ index (a readmission risk prediction index based on length of stay, acuity of admission, comorbidities, and emergency department visit history) between 50 and 75 [7]. Patients with LACE+ scores outside this range could be referred at provider discretion. Patients were excluded from appointment scheduling if they were discharged to a care facility or hospice or had an existing PCP appointment within 7 days of discharge.

The virtual clinic was supported by 12 hospitalists, 2 medical assistants, 1 pharmacist and an on-demand interpreter service. Medical assistants contacted eligible patients to schedule web-based video visits. VToC visits were occasionally converted to telephone encounters when patients faced technical barriers, which occurred in fewer than 5% of cases and thus were not considered a distinct comparison group for analysis. Patients received phone call reminders 1 business day prior to scheduled appointments. Pharmacists conducted previsit medication reviews to identify fill history gaps, dosing inconsistencies, and potential drug-drug interactions. Note templates guided VToC providers in addressing medication management, social determinants of health, barriers to follow-up, and care coordination. A standardized electronic handoff was routed to the patient’s PCP and relevant specialists summarizing the reason for hospitalization, follow-up needs, and timing recommendations. Additionally, post-VToC visit communication was sent to the discharging hospitalist to highlight potential care lapses and support quality improvement efforts.

Data were collected from the electronic health records using standard SQL queries. These included demographics (ages grouped as <45, 45-64, and ≥65 years; sex; and race and ethnicity); VToC status (yes or no); PCP (University of California San Diego or other); LACE+ score (<50, 50-75, or >75); and 30-day all-cause readmission. Patient characteristics were analyzed by VToC status (yes or no). A VToC status of “no” indicates patients who were discharged from the medicine service line to home and not seen in our clinic (because they did not meet eligibility criteria or could not be reached). Patients were excluded from analysis if they had missing demographic or clinical data. Readmission rates were analyzed for VToC status and by LACE+ score. Chi-square analysis was used to compare characteristics by VToC status with a *P*<.05 considered significant. Readmission rates (%) by VToC status stratified by LACE+ decile are reported. Error bars were calculated using 95% CIs. A multivariable logistic regression analysis was performed to assess independent associations with unplanned hospital readmission. Variables included in the model included age group, sex, race and ethnicity, PCP affiliation (UCSD versus non-UCSD), LACE+ score, and VToC enrollment. All analyses were conducted using the SPSS Statistics software suite (version 27.0; IBM Corporation).

### Ethical Considerations

This study was conducted in an Institutional Review Board–exempt fashion in accordance with the ethical standards of the University of California San Diego Human Research Protection Program and the Office for Human Research Protections and adhered to the Helsinki Declaration of 1975, as revised in 2000.

## Results

There were 25,970 patients identified for the study period of September 1, 2021, through September 17, 2024. A total of 527 (0.02%) patients had missing data and were excluded from the analysis. Of the 25,443 patients included in the study, 2314 (9.1%) were seen in VToC after discharge and 23,129 (90.9%) had standard of care follow-up. Table 1 shows the demographic comparisons of patients who were seen in VToC versus those who were not for the same time period.

**Table 1:** Demographics of virtual transitions of care (VToC) and non-VToC patients (N=25,443).

		VToC (n=2314)	Non-VToC (n=23,129)	Total (N=25,443)	*P* value
**Age range, (y), n (%)**					.40
	<45	558 (24.1)	5829 (25.2)	6387 (25.1)	
	45-64	861 (37.2)	8325 (36)	9186 (36.1)	
	≥65	895 (38.7)	8975 (38.8)	9870 (38.8)	
**Sex, n (%)**					<.001
	Female	1266 (54.7)	10,943 (47.3)	12,209 (48)	
	Male	1048 (45.3)	12,186 (52.7)	13,234 (52)	
**Race or ethnicity, n (%)**					<.001
	Non-Hispanic White	870 (37.6)	9795 (42.3)	10,665 (41.9)	
	Hispanic	834 (36)	7199 (31.1)	8033 (31.6)	
	Non-Hispanic Black or African American	213 (9.2)	2171 (9.4)	2384 (9.4)	
	Non-Hispanic Asian, American Indian, or Alaskan Native	205 (8.9)	1949 (8.4)	2154 (8.5)	
	Other^a^ or unknown	192 (8.3)	2015 (8.7)	2207 (8.7)	
**PCP^b^, n (%)**					.75
	UCSD^c^ PCP	644 (27.8)	6509 (28.1)	7153 (28.1)	
	Non-UCSD PCP	1670 (72.2)	16,620 (71.9)	18,290 (71.9)	
**LACE+ score range, n (%)**					<.001
	<50	79 (3.4)	2321 (10)	2400 (9.4)	
	50-70	113 (65.4)	9152 (39.6)	10,665 (41.9)	
	>70	722 (31.2)	11,656 (50.4)	12,378 (48.6)	

^a^Self-reported “other” race or ethnicity or missing data.

^b^PCP: primary care provider.

^c^UCSD: University of California San Diego.

**Figure 1:**
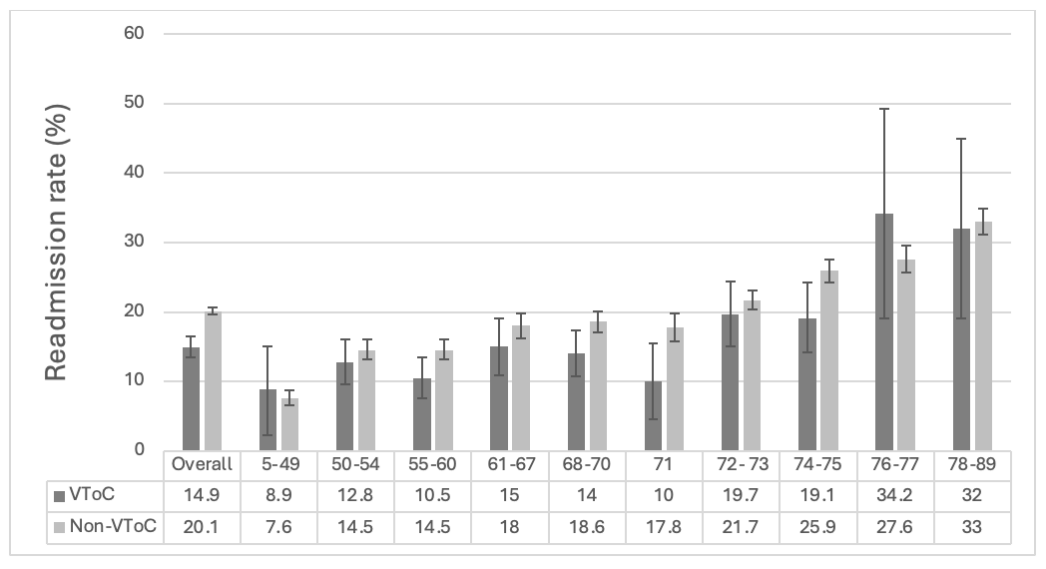
This figure presents 30-day readmission rates for patients who received the virtual transitions of care (VToC) intervention (dark bars) versus those who did not (light bars), stratified by LACE+ score decile groups. The x-axis represents different LACE+ decile groups, while the y-axis shows readmission rates as percentages. Error bars are included for each proportion and represent the difference between the point estimate (proportion) and upper or lower CI. The targeted population for this intervention was patients with LACE+ scores between 50 and 75. Deciles outside this range are included for completeness. Across most LACE+ decile ranges, the VToC group consistently showed lower readmission rates compared to the non-VToC group.

The overall 30-day readmission rate for patients seen in the VToC clinic was 14.9% (344/2314) compared to 20.1% (4659/23,129) in the benchmark group (Table 1; *P*<.001). In the regression model, the absence of a VToC clinic was independently associated with increased odds of readmission (odds ratio 1.37; 95% CI 1.21-1.54; *P*<.001). In the multivariable regression analysis, younger age, female sex, non-Hispanic Asian race or ethnicity, higher LACE+ scores, and non-participation in the VToC program were each independently associated with increased odds of hospital readmission." should be changed to "In the multivariable regression analysis, younger age, female sex, Hispanic and Non Hispanic Black/African American race or ethnicity, higher LACE+ scores, and nonparticipation in the VToC program were each independently associated with increased odds of hospital readmission. This analysis is available in Multimedia Appendix 1. Decile matching was performed to account for the difference in distribution of LACE+ in the VToC and non-VToC groups. Analysis of LACE+ decile groups revealed that the impact of readmission reduction was most prominent among patients with LACE+ scores ranging between 50-75; however, across most groups VTOC performed better than non-VToC (Figure 1).

## Discussion

Here we present a hospitalist-led postdischarge virtual clinic aimed at improving transitions of care for patients. For patients at moderate risk, a VToC visit was associated with a significantly lower rate of readmission. Notably, our intervention group had a higher proportion of female and Hispanic patients, both of which were associated with increased odds of readmission in regression analyses.

This observational study is subject to selection bias, as clinic attendance could be influenced by patient-specific factors that influence readmission. Approximately 13% of patients referred to the VToC were not reached, which may introduce additional bias, as these individuals may differ in ways that influence outcomes. While we did not capture detailed data on patient acceptance, we addressed this limitation by matching the comparator group on readmission risk to reduce confounding. However, the stratified analysis according to the LACE+ index provides strong evidence in support of the impact of the program, and the positive impact observed is consistent with broader evidence that timely postdischarge access reduces readmission risk. VToC clinics address a critical gap in the care continuum while also providing the inherent benefits of telemedicine [6,8].

Results from this initiative are promising for the feasibility of telemedicine-based transitional care programs to promote the quadruple aim of improving population health, enhancing the care experience, reducing cost, and advancing care equity [9].

## Appendix

Multimedia Appendix 1 Supplementary data.

## Abbreviations

PCP: primary care provider
VToC: virtual transitions of care

## Data Availability

All data generated or analyzed during this study are included in this published article and its supplementary information files.

## References

[1] Barbut D, Borer JS, Wallerson D, Ameisen O, Lockshin M (1991). Anticardiolipin antibody and stroke: possible relation of valvular heart disease and embolic events. Cardiology.

[2] Anderson A, Mills CW, Willits J, Lisk C, Maksut JL, Khau MT, Scholle SH (2022). Follow-up post-discharge and readmission disparities among Medicare fee-for-service beneficiaries, 2018. J Gen Intern Med.

[3] Zhang X, Lin D, Pforsich H, Lin VW (2020). Physician workforce in the United States of America: forecasting nationwide shortages. Hum Resour Health.

[4] Baldino M, Bonaguro AM, Burgwardt S, Lombardi A, Cristancho C, Mann C, Wright D, Jackson C, Seth A (2021). Impact of a novel post-discharge transitions of care clinic on hospital readmissions. J Natl Med Assoc.

[5] Wiest D, Yang Q, Wilson C, Dravid N (2019). Outcomes of a citywide campaign to reduce Medicaid hospital readmissions with connection to primary care within 7 days of hospital discharge. JAMA Netw Open.

[6] Shah DA, Sall D, Peng W, Sharer R, Essary AC, Radhakrishnan P (2024). Exploring the role of telehealth in providing equitable healthcare to the vulnerable patient population during COVID-19. J Telemed Telecare.

[7] van Walraven C, Wong J, Forster A (2012). LACE+ index: extension of a validated index to predict early death or urgent readmission after hospital discharge using administrative data. Open Med.

[8] Reeves JJ, Ayers JW, Longhurst CA (2021). Telehealth in the COVID-19 era: a balancing act to avoid harm. J Med Internet Res.

[9] Nundy S, Cooper LA, Mate KS (2022). The quintuple aim for health care improvement: a new imperative to advance health equity. JAMA.

